# Nitrogen-Doped Graphene Oxide as Efficient Metal-Free Electrocatalyst in PEM Fuel Cells

**DOI:** 10.3390/nano13071233

**Published:** 2023-03-30

**Authors:** Adriana Marinoiu, Mircea Raceanu, Elena Carcadea, Mihai Varlam

**Affiliations:** 1 ICSI Energy Department, National Research and Development Institute for Cryogenic and Isotopic Technologies, 240050 Ramnicu Valcea, Romania; 2 Doctoral School, University Politehnica of Bucharest, 060042 Bucharest, Romania

**Keywords:** electrocatalyst, nitrogen-doped graphene oxide, metal-free, long-term operation stability, oxygen reduction reaction, proton-exchange membrane fuel cells

## Abstract

Nitrogen-doped graphene is currently recognized as one of the most promising catalysts for the oxygen reduction reaction (ORR). It has been demonstrated to act as a metal-free electrode with good electrocatalytic activity and long-term operation stability, excellent for the ORR in proton exchange membrane fuel cells (PEMFCs). As a consequence, intensive research has been dedicated to the investigation of this catalyst through varying the methodologies for the synthesis, characterization, and technologies improvement. A simple, scalable, single-step synthesis method for nitrogen-doped graphene oxide preparation was adopted in this paper. The physical and chemical properties of various materials obtained from different precursors have been evaluated and compared, leading to the conclusion that ammonia allows for a higher resulting nitrogen concentration, due to its high vapor pressure, which facilitates the functionalization reaction of graphene oxide. Electrochemical measurements indicated that the presence of nitrogen-doped oxide can effectively enhance the electrocatalytic activity and stability for ORR, making it a viable candidate for practical application as a PEMFC cathode electrode.

## 1. Introduction

It is a common belief that nowadays we are witnessing an extremely exciting progress for the fuel cells (FCs), mainly due to the recognition of hydrogen as an attractive energy storage technology [1]. One promising solution represents the proton exchange membrane fuel cell (PEMFC) technology, which can definitely become a sustainable and clean electricity source. Light vehicles or stationary applications are just a few examples of applications where low temperature fuel cells can be successfully used.

However, the cost and durability of PEMFCs are still the main hindrances disfavoring the full commercialization on a large scale. The durability decreases mainly due to the degradation of FC components, the most important being the electrodes. It is known that the degradation rate at the cathode-side is higher than that of the anode side and substantially affects the outcomes of the PEMFC.

In addition, the key contributors to FC degradation are the following: the carbon support corrosion, loss of catalytic surface area, and deterioration of the membrane electrode assembly. The specific operation conditions inevitably generates a slow kinetic of the oxygen reduction reaction (ORR) at the cathode. The cathode degradation is the most significant, thus the development of a highly durable cathode has become a subject of great interest. The commonly used catalyst in PEMFC is platinum (Pt) due to its good catalytic activity. However, the limited abundance, high cost of Pt, as well as the stability and resistance to corrosion in the operating environment involved considerable efforts to identify some alternatives for Pt catalyst, but without diminishing the electrochemical performances.

The research strategy to obtain a high-performance and less expensive ORR catalyst consisted in two main approaches. The first direction was to diminish the metal catalyst loading by improving the platinum’s utilization grade by (i) alloying Pt with less expensive transitional metals (Fe, Ni, Co, so on) or (ii) considering different support material for the deposition of noble metal particle, such as various types of carbon (graphene-based materials, carbon nanotubes). The second direction was to use the platinum group metals (PGMs) and the PGM-free materials. The actual state-of-the-art (SoA) research indicates that addressing a durable performance metal-free catalyst for oxygen reduction reaction (ORR) could significantly influence the widespread commercialization of PEMFC-based applications such as hybrid vehicles [2,3,4,5].

On the other hand, the most effective use of catalyst is possible only by considering a good material with high specific surface area, which can supply as many binding sites, where the catalyst nanoparticles could be anchored. Extended efforts were dedicated to various carbon nanomaterials such graphene-based materials, carbon nanofibers, or nanotubes, as support for anchoring the catalyst nanoparticles. Through innovative nanomaterials, graphene-based materials have stood out as a remarkable support in catalysis, by considering the high specific surface area, electrical conductivity, and interesting mechanical and chemical stability in the specific PEMFC operating conditions. Among graphene materials, graphene oxide (GO) has received remarkable attention as a valuable class of graphene derivatives, due to its chemical stability, high conductivity, and ability to form chemical bonds easily [6,7,8,9].

Recent research has shown that the introduction of heteroatoms into the carbon lattice could significantly improve surface properties, mainly by optimizing the electronic charge distribution. Thus, doping with p and n type elements (N, B, O, P, S) could be an efficient way to modify the electrical, chemical, or physical properties of GO. Through the different heteroatoms evaluated for functionalization/doping, the nitrogen has received a significant interest, especially due to the fact that it possesses the same atomic size as carbon, having one more electron than carbon. Thus, the electronic distribution of carbon atoms is disturbed when nitrogen is introduced into the graphene array.

Up to now, the nitrogen-doped graphene oxide (N/GO) represents one of the promising non-platinum catalysts mainly due to the advantages of low cost and high catalytic performance to reduce/replace the expensive Pt-based catalysts in various electrochemical devices [10,11,12]. Nitrogen doping disrupts the ideal sp^2^ hybridization of carbon atoms and significantly changes the chemical reactivity and electronic properties of GO [13,14,15]. In particular, N/GO has been extensively investigated as a promising catalyst for the oxygen reduction reaction (ORR), especially due to improved electronic properties in the electron transfer reactions.

Significant progress has been noted with respect to the evaluation of non-PGM materials considering the development and improvement of innovative carbon structures, like nitrogen-doped carbon. Recently, the N/GO was demonstrated to act as metal-free electrode with a better electrocatalytic activity, long-term operation stability for oxygen reduction via a four-electron pathway in PEM fuel cells. Even the exact catalytic mechanism is still under debate, N/GO catalyst has demonstrated remarkable results for ORR and a high stability for FCs. Moreover, it has been noted that N/GO catalysts provided a catalytic activity comparable to that offered by Pt-based catalysts [15,16].

Thus, many papers were dedicated to the investigation of this catalyst through the methodologies for the synthesis, characterization, and technologies improvement [17,18,19,20,21,22,23].

Investigations about different chemical, physical, and mechano-chemical preparation procedures were discussed based on the features of N/GO catalysts, in particular regarding the nitrogen content. The literature presents various published papers for the synthesis of N/GO. Various substances, such as ammonia, urea, ammonium salts, and organic salts have been investigated as precursors of N. Until recently, N-doped graphene was prepared by using one of the techniques presented below: chemical vapor decomposition (CVD), the discharge of a graphite electrode in the presence of pyridine vapor, thermal exfoliation of graphite oxide with NH_3_, plasma nitrogen treatment of graphene, thermal decomposition, and solvothermal pathway [24].

The chemical vapor deposition (CVD) is the classic route to prepare the doped-graphene or N/GO catalysts for industrial and lab scale. However, this method involves several important disadvantages, namely: introduction of metal impurities (e.g., from Cu or Ni support) in the final doped graphene; using of gaseous ammonia as nitrogen source is a very toxic process, thus the additional costs are necessary to meet the special safety treatments. In this context, a metal-free route is recommended to be developed for the preparation of N/GO materials [15,25,26].

The CVD process synthesized N/GO directly on copper foil substrate, using urea, boric acid, and polystyrene as precursors containing carbon and nitrogen. Modifying the concentration of precursors, the nitrogen-doped concentration can be adjusted from 0.9% to 4.8%. It was noted that different types of nitrogen could be obtained. Graphene doped with about 2.1% indicated that N bond configuration was predominated by pyridine N. However, for graphene doped with 4.8%, the final product consists of 60.2% N-pyridine content and 39.8% N-pyrrole content. These results specified that the characteristics of N/GO could be optimized and controlled by modifying the degree of doping [27]. However, the state-of-the-art nitrogen doping level in N/GO involving the CVD process is between 3 wt.% to 8 wt.% [16,28].

The heat treatment method was involved to prepare N/GO by using a solid carbon source (e.g., graphene oxide), a nitrogen source (e.g., melamine), and a high temperature during the heat treatment (between 500–1000 °C). Apart from the obvious disadvantage of the high temperatures, it should be mentioned that it is necessary to use argon gas to secure the environment in which the carbon nitride polymer is produced. Moreover, it is very difficult to control the final nitrogen content at the different temperatures used during the process, which really hinders the large-scale development [23,29].

To date, N/GOs have been predominantly synthesized by various methods of thermal treatment. A catalyst-free synthesis method has been developed for the preparation of N/GO by heat treatment of graphite oxide with melamine (as a nitrogen source). The method included three steps: (i) the melamine molecules were adsorbed on pre-synthesized graphene oxide (GO); (ii) the melamine was converted to carbon nitride at elevated temperatures (above 500 °C); (iii) in the end, once the oxygen groups are removed from the graphene nanoparticles at high temperatures, the N atoms could dope the network. The concentration of doped nitrogen can be optimized by modifying the mass ratio between GO and melamine or by varying the temperature. The highest nitrogen content was 10 wt.% corresponding to a ratio GO to melamine of 1:5 (calculated as mass ratio) obtained at 700 °C. N/GO was prepared by thermal hardening of GO in an ammonia atmosphere. Oxygen-containing groups on the GO surface have been shown to be fundamental for C-N bond obtaining during the reaction with GO and NH_3_. It is important to note that the biggest N content of 5 wt.% has been obtained at temperature of 500 °C.

Latterly, a synthesis method in gaseous phase with graphene substrate has been performed by considering the plasma process. The nitrogen atoms can partially replace carbon atoms from graphene structure by using the N_2_ or NH_3_ plasma treatment [29]. Gas annealing method is similar with the previous method in respect to the heat treatment. The method uses a solid carbon source (e.g., graphene oxide, carbon black) and nitrogen source (e.g., ammonia) and a high temperature. The main disadvantage of this route is the huge amount of inert gas used for many hours in the tubular furnace to secure the obtained N/GO from the air [30].

Even the reaction occurs as a low time-consuming method (up to 1 h), however the plasma treatment during the reaction involves some special instrumental requirements for nitrogen plasma generating, namely a high power and pressure, thus not favoring the scaling of the process at commercial scale [31].

The percentage of N atoms introduced in the GO structure could be controlled in the domain of 0.11–1.35 wt.%, especially by optimizing the exposure time. Taking into account this aspect, the use of plasma protocol not only led to the including of N into the graphene network, but to increasing the oxygen content too, from approx. 15 wt.% in the case of graphene, up to approx. 27 wt.% in N/GO. A step-by-step approach was attempted to reduce GO and to introduce nitrogen in GO network at the same time at room temperature, by involving of plasma treatment in an atmosphere of a H_2_ and NH_3_ gas mixture. Through this chemical process, the highest nitrogen concentration of about 5.8 wt.% can be obtained, but the process still remains at the small scale [27,28,29,30,31,32].

The nanoscale high-energy wet ball milling derives from the previous method and results in minimal contamination, but involves more difficult operating conditions, thus the method is difficult to scale-up commercially.

A series of solvothermal and hydrothermal treatments for the preparation of N/GO have been developed in the presence of cyanuric chloride (N_3_C_3_Cl_3_), Li_3_N, and CCl_4_ at 350 °C, for 6 h [32].

According to the SoA approaches for producing of N/GO, the conventional chemical methods such as CVD or thermal treatment have been considered more or less standard for the preparation of N/GO-based materials. The main disadvantages of the mentioned processes are mentioned: the preparation methods involved require multiple operating activities and sophisticated equipment, making the processes unattractive to be transposed to a larger production scale. Other disadvantages are harsh conditions requiring elevated temperatures for thermal decomposition and high pressure or supercritical conditions. Furthermore, these processes result in poorer quality graphene materials (many structural defects). The preparing of N/GO using a simple synthesis method is still a topical issue in this context.

The current study introduces an original protocol based on a low time-consuming method to obtain N/GO, considering a commercial graphene oxide, different nitrogen precursors (ammonia, urea, nitric acid) and different reducing agents.

## 2. Experimental

The nitrogen-doped graphene oxide (N/GO) samples were obtained starting from commercial graphene oxide by a one-step reaction process, under mild reaction conditions in the microwave field (60–80 °C, 800 W). The obtained materials were analyzed by different compositional, morphological, and structural characterization techniques and various electrochemical methods.

### 2.1. Materials

Graphene oxide powder (medium reduced GO, product no.2.1-M) was supplied by Abalonix Norway. Ammonia (25 wt.%) was purchased from Chimreactiv Romania; urea (98 wt.%), nitric acid (65 wt.%), ethanol and ethylene glycol were acquired from Alfa Aesar; Sharlau delivered synthetic grade sodium borohydride.

### 2.2. Catalyst Preparation

An amount of 250 mg of graphene oxide (GO) powder was well dispersed in demineralized water and then in 100 mL of reduction agent solution (ethylene glycol EG, sodium borohydride BH_4_Na, ethanol Et), using both an ultrasonic bath and an ultrasonicator as well. Ammonia solution was added to the GO dispersion and sonicated again. The resulting mixture was placed in a microwave reactor (MARS 6 One touch oven, EMC) for 15 min under the following reaction conditions: reaction temperature 60–80 °C, microwave power 800 W. The reaction mixture, containing a polar liquid, absorbs microwave energy very quickly. Thus, the temperature in the suspension mass increases rapidly, causing the reaction of the sample in a short time. The reaction product was removed separately, washed well with deionized water and finally lyophilized. The obtained product (N/GO_A) in powder form was perfectly dispersible in deionized water (ultrasonic bath, 15 min) and could be dried and redispersed, being suitable for catalytic material.

A second route to obtain the nitrogen-based graphene oxide is presented. An aqueous solution based on urea (2.43 wt.%) was used as a nitrogen source, completely non-toxic and non-flammable. Nitrogen-doped graphene was prepared using a simple chemical method, described below. An amount of 250 mg of graphene oxide (GO) powder was well dispersed in demineralized water and then in ethanol solution, using both an ultrasonic bath and an ultrasonicator. The urea solution (2.5 g urea 98 wt.%, 100 mL deionized water) was introduced in the GO-based dispersion, and then ultrasonicated again. The obtained mixture was introduced into the microwave reactor, under the mentioned reaction conditions (reaction temperature 60–80 °C, microwave power 800 W, reaction time 15 min). The reaction product was separated, washed thoroughly with deionized water and finally dried by lyophilization. N/GO_U powder was obtained, as a product also perfectly dispersible in water.

A third example of nitrogen-based graphene oxide is presented. A dispersion of GO (250 mg powder in 100 mL demineralized water) was added to 100 mL of reduction agent solution (sodium borohydride BH_4_Na), using both ultrasonic bath and ultrasonicator. Nitric acid solution was added to the GO-based dispersion and sonicated again. The resulting mixture was inserted into a cell made of Teflon of the MARS 6 One touch reactor. The reaction conditions were mentioned above. The product obtained was separated by ultracentrifugation, washed with deionized water and finally subjected to lyophilization. The final powder-like N/GO_N product was evaluated as a catalytic material.

### 2.3. Characterization Methods

X-ray photoelectron spectroscopy was carried out by the X-ray photoelectron spectrometer (PHI-5000 VersaProbe, PHI-Ulvac/Physical Electronics, Chigasaki, Japan). XPS measurements were obtained using the monochromatic Al Kα radiation (1486.7 eV). The photoelectrons were collected under an angle of 45°. The elemental qualitative analysis was determined by recording the broad spectra and then identifying various chemical bindings on the surface; finally, the deconvolutions of high resolution spectra were performed. XPS curves were interpreted from the PHI-MultiPak software. The atomic concentrations of the various chemical elements were calculated, considering the sensitivity factors for the elements identified on the surface, thus achieving a quantitative elemental analysis. The specific surface area measurements of graphene doped with nitrogen (powder) were performed using the Autosorb IQ Quantachrome equipment by the Brunauer–Emmett–Teller (BET) method. The nitrogen adsorption and desorption isotherms were measured at 77 K, and the specific surface area was calculated. The porosity analysis, the pore volume as well as the pore radius were estimated by considering the Barret–Joyner–Halenda method (BJH). Before the actual adsorption measurements, the prepared graphene-based materials were degassed at 393 K, time for 10 h. The elemental analysis was involved for a rapid and accurate analysis of solid materials to provide the C, H, N, and O concentrations. The TGA-DSC STA 449 Jupiter Simultaneous Thermal Analyzer (TG-DSC) with operating range between 25–1650 °C and controlled argon atmosphere was used to perform the simultaneous analyses of thermogravimetric mass loss (TG) and differential scanning calorimetry (DSC). For the quantification of carbon, nitrogen, and hydrogen, the combustion method at 950 °C was used, while the pyrolysis process at 1050 °C was carried out for oxygen calculation. The gas was separated by using the SM5A column (molecular sieve) and also detected by the TCD. The IR spectra (KBr pellets form) were collected on a Bruker Tensor 37 spectrometer in the range 4000–400 cm^−1^.

*Electrode Preparation and Electrochemical Measurements*

The ex situ electrochemical analysis was performed by using the VersaSCAN electrochemical workstation (Princeton Applied Research) through the following electrochemical methods: the cyclic voltammetry (CV) and electrochemical impedance spectroscopy (EIS). CV measurements were mainly carried out to evaluate the electrochemical response of prepared N/GOs in term of stability. Therefore, a catalytic ink was prepared by making a suspension of the obtained N/GOs samples in C_3_H_7_OH (Alfa Aesar, Haverhill, MA, USA), deionized water and 5 wt.% Nafion solution (Aldrich, St. Louis, MO, USA) and then sonication for 3 h. The working electrode (WE, 5 mm diameter, 0.196 cm^2^) was carefully polished with 0.3 and 0.05 mm alumina slurry, respectively, and then ultrasonicated in ethanol and deionized water several times to obtain an almost perfect mirror-like surface. The electrode was obtained through deposition on pretreated WE of 5 μL from the as prepared catalytic ink, then the electrode was dried in an oven (at about 40 °C for 15 min). After evaporation of the solvent, the deposited catalyst was covered with 5 mL Nafion solution (0.5 wt.% in ethanol) to fix the catalyst on the surface. The prepared electrodes were allowed to dry in air. The conventional three electrodes electrochemical system (Ag/AgCl electrode and platinum wire as the reference and counter electrode, respectively) was used. The electrolyte was 0.1 M KOH. The CV potential was evaluated between −0.8 V to 0.3 V, using various voltage scan rates (25, 50, 75 and 100 mV s^−1^). EIS measurements were evaluated in the frequency domain of 0.01 Hz to 100 kHz (in alternating AC voltage of 10 mV).

The in situ electrochemical analysis was performed by using the home-made FC workstation, composed by: DS electronic load, bubble-type humidifiers. The single FC was operated at 0.6 V for 1 h for MEA conditioning. A test cell hardware and compression set designed from Fraunhofer ISE for Baltic Fuel Cells GmbH (Karlsruhe, Germany) was used for compression with a pneumatic cylinder. The compression force (1.2 N mm^−2^) was exclusively on the active area (50 × 50 mm) and not on the seals (no seals on the CCM, but a radial seal on the compression cylinder). The clamping pressure was controlled by a pneumatic cylinder so that the cell compression was independent of the GDL thickness, a ball joint leads to a homogeneous compression. To reduce boundary effects, the GDL overlaps the CCM area. The temperature sensor was placed in the cooling field, liquid cooling being applied. Copper parts were used for good heat conduction, but to avoid corrosion problems, these parts are gold plated. The geometry of the flow channels grooved in the bipolar plates was serpentine type for both. The humidified gases were introduced at 60–70 °C in countercurrent—flow pressure (atmospheric and applied back pressure of 1 bar). Gas flow rates (H_2_ fed to anode, air to cathode, and N_2_ as carrier) were fixed by using flow controllers. A control system based on NI c-RIO hardware was involved to control the workstation. The fuel cell was operated at 0.6 V for 1 h for membrane electrode assembly conditioning.

## 3. Results and Discussion

A successful synthesis protocol based on microwave method is proposed in this paper to prepare nitrogen-doped graphene oxides (N/GOs). This was confirmed and validated by using a chemical, physical, and electrochemical investigation. The electro-catalytic characteristics of the prepared N/GOs samples are directly connected to the chemical structure, mainly to the elemental composition and also to the chemical bonding. The experimental conditions influenced the nitrogen doping ratio, mainly by nitrogen precursors, both organic and inorganic compounds.

The nitrogen doping could be usually attained through rather severe chemical methods, such as CVD of nitrogen-containing precursors, high-temperature ammonia treatment with GO or solvothermal process of GO with nitrogen containing atoms, followed by annealing at elevated temperatures.

This study explores the challenge to obtain N/GOs under mild reaction conditions, in a microwave (MW) field, starting from different nitrogen precursors. The protocol could be a challenge for controlling the doping level, when different types of nitrogen centers were going to be inserted into the graphene network. Ammonia was used as a first nitrogen source, this precursor being well known in N1 chemistry. The ammonia molecule has a pyramidal-trigonal structure, with tetrahedral surfaces, which have a nitrogen atom with a free pair of electrons in the corner. Ammonia has an amphoteric character and contains a large amount of nitrogen (82% by mass) and a bifunctional structure. Urea (also called carbonic acid diamide) is the organic compound with the molecular formula CO (NH_2_)_2_, frequently used in organic reactions, due to its high N content. Nitric acid is the primary reagent used for nitration typically in an organic molecule and was the third nitrogen precursor in this study.

The chemical composition of the prepared samples was investigated primarily by the elemental analysis using the combustion method (for C, H, N) and the pyrolysis method (for O). The results indicated the presence of all mentioned elements and the chemical composition is provided in Table 1 and Table 2.

Even the presence of nitrogen in all obtained materials was noted, however the difference of nitrogen content was generated by different precursors as well as various reduction agents. This indicated that the highest concentrations of nitrogen were obtained when ammonia and urea were used as precursors. An important conclusion derives from the fact that, even if the functionalization of graphene oxide during microwave process does not require a high pressure or a high temperature, still the heating temperature could be one of the factors that significantly influences the nitrogen content in synthesized catalysts.

The physical and chemical properties of the materials obtained from different precursors (such as ammonia, urea and nitric acid) were evaluated and compared. It was found that ammonia allows for a higher concentration of nitrogen, probably due to its high vapor pressure which facilitates the reaction of GO functionalization during microwave. Thus, it has been shown that the microwave can grind easily and even break the bulk solid powder to chop particles. The microwave reaction occurs in a liquid environment, so the phenomena of mechanical breakage and cavitation-related effect and shock waves are limited. This type of mechanism acts in process intensification to increase the reaction rates or production yields due to its direct (mechanical, thermal, and chemical) and secondary effects, including turbulence, mechanical vibration, macroscopic heating, emulsification, and dispersion [33].

One material from each group was selected, namely the material with the highest nitrogen concentration, and their physico–chemical and electrochemical characterizations will be presented below. The samples were encoded: N/GO_A obtained from ammonia as nitrogen precursor, N/GO_U obtained from urea as nitrogen precursor, N/GO_N obtained from nitric acid.

**Figure 1:**
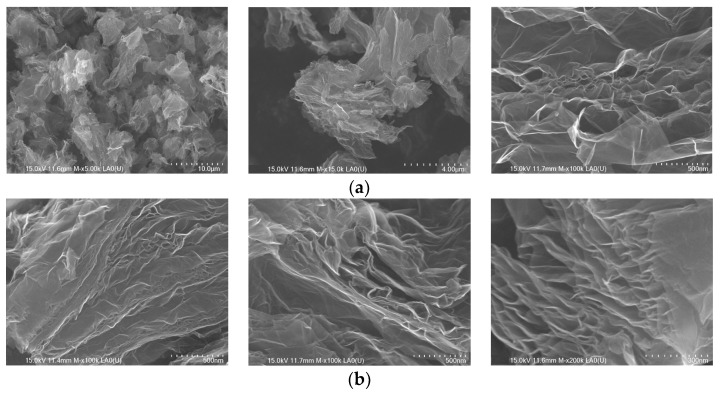
(**a**) SEM images of graphene oxide (GO); (**b**) SEM images of nitrogen-doped graphene oxide materials prepared by using different nitrogen precursors: N/GO_A (left)—from ammonia; N/GO_U (middle)—from urea, and N/GO_N (right)—from nitric acid.

**Figure 2:**
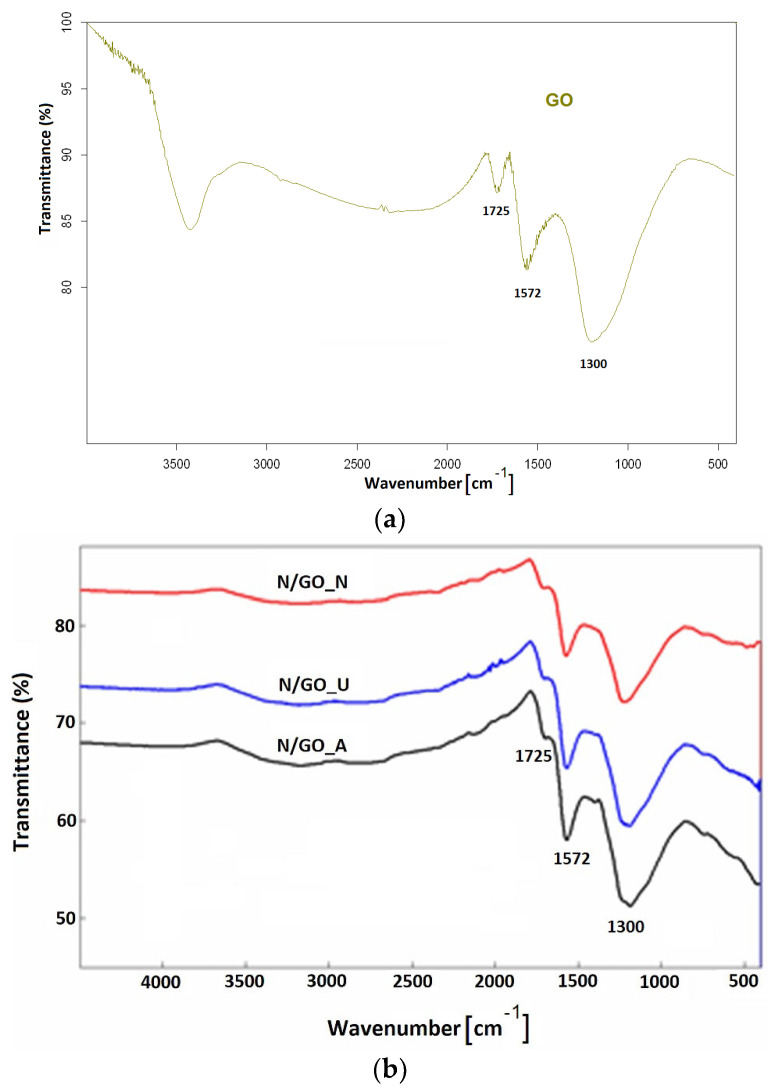
FT−IR spectra of: (**a**) GO and (**b**) Nitrogen-doped graphene oxide materials prepared by using different nitrogen precursors: N/GO_A from ammonia; N/GO_U from urea, and N/GO_N from nitric acid.

Figure 1a,b reveals the SEM micrographs of the bare graphene oxide and prepared nitrogen-doped graphene oxide samples. The SEM micrographs of the bare graphene oxide in Figure 1a reveals the wavy, corrugated, and wrinkled morphology. However, Figure 1a (left and middle) appears to have fewer similarities. Particularly, Figure 1a (left) does not seem to match any of the figures in Figure 2. This implies that not all features of the basic structure remain intact in the nitrogen-doped graphene oxide. Figure 2b has an actual resemblance only to the pattern of Figure 1a (right): the highest match for N/GO_N and the least for N/GO_A. This means that the higher content of N%, in fact, changes the morphology of the basic structure of GO.

Fourier transform infrared spectroscopy (FT-IR) was involved to investigate the functional groups on the surface of the prepared N/GOs. FT-IR spectra shown in Figure 2 indicate mainly, the evidence of oxygen containing functional groups in all samples. The main characteristic bands were assigned: intense peaks at approx. 1572 cm^−1^ were assigned to the skeletal vibrations from non-oxidized graphitic domains from aromatic regions of GO; small peaks at around 1725 cm^−1^ correspond to C=O stretching and indicates the fact that this bond almost disappeared after microwave treatment. According to the literature, the identification of chemisorbed nitrogen on the GO surface is hindered by its spectral similarity to epoxy oxygen, which usually exists in the graphene lattice, particularly for materials obtained by reduction of GO. Thus, the graphene sp^2^ configuration could restrain the graphitic N confinement because the nitrogen atom has almost the same atomic radius as the carbon atom. The identification is even more complicated since numerous feasible overlapping vibrational modes exist in the 1150−1600 cm^−1^ domain. However, a sharp peak at approx. 1300 cm^−1^ was clearly identified in the spectrum of N/GO synthesized through the pyrolysis of GO and urea, when the authors hypothesized that N was chemisorbed on the carbon nanotubes walls and assigned these peaks to C−N and N−CH_3_ vibrations and pyridinic-N configuration [34]. In general, the pyridinic N bonds with two C atoms at the edges or defects of graphene and could contribute with one *p* electron to the π system.

However, IR spectroscopy is not appropriate to distinguish between the different valence states of nitrogen in N/GO, mainly because the specific vibrations of nitrogen in N/GO materials are coupled with additional vibrations of carbon atoms coming from some symmetry changes induced by the doping process [34].

Regarding the prepared N/GOs, it is important to note the preservation of some oxygen functionalities from the initial GO structure namely the carboxyl group, which increase the conductivity of the doped material, providing an advantage for the application as electrodes for energy storage devices.

In contrast to the FTIR method, the X-ray photoelectron spectroscopy (XPS) represents the most appropriate spectroscopic method to remark and highlight between the different nitrogen bonds in graphene-based materials, especially since the various forms of N provide significantly different binding energies. The elemental composition of N/GOs was achieved by XPS measurements. The qualitative analysis of the chemical elements linked to the GO surface was investigated after obtaining the broad spectra, and the identification of various types of chemical bonds formed on the surface during the chemical reaction, was achieved by deconvolution of the high-resolution spectra of the existing chemical elements.

**Figure 3:**
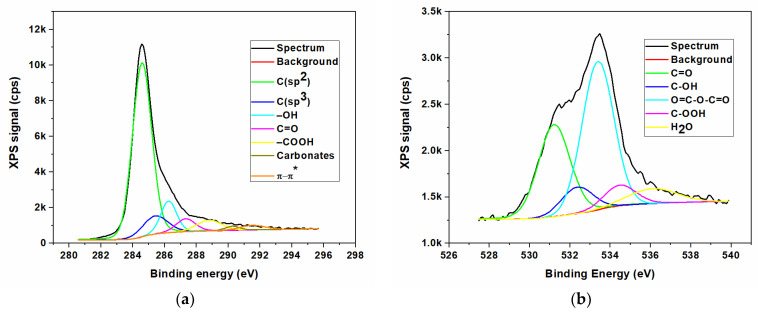
XPS high resolution spectra for non-doped graphene oxide (GO); (**a**) Carbon (C1s); (**b**) Oxygen (O1s).

**Figure 4:**
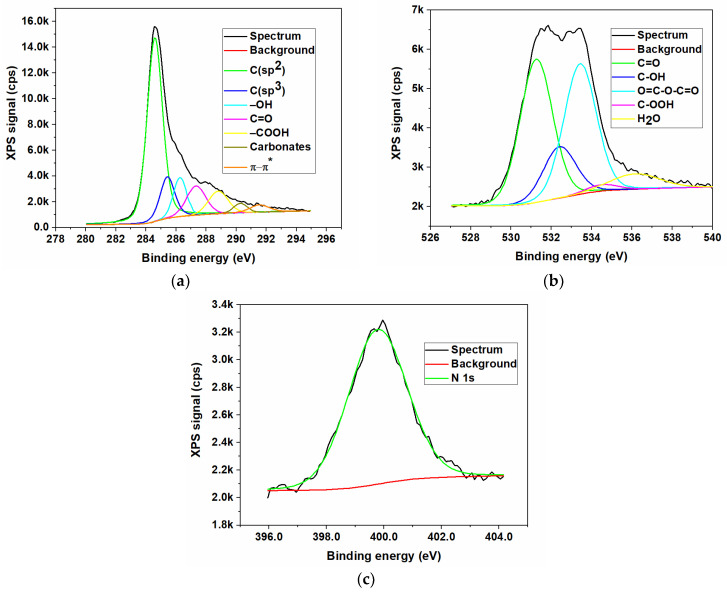
XPS high resolution spectra for nitrogen-doped graphene oxide synthesized using ammonia (N/GO_A); (**a**) Carbon (C1s); (**b**) Oxygen (O1s); (**c**) Nitrogen N1s.

**Figure 5:**
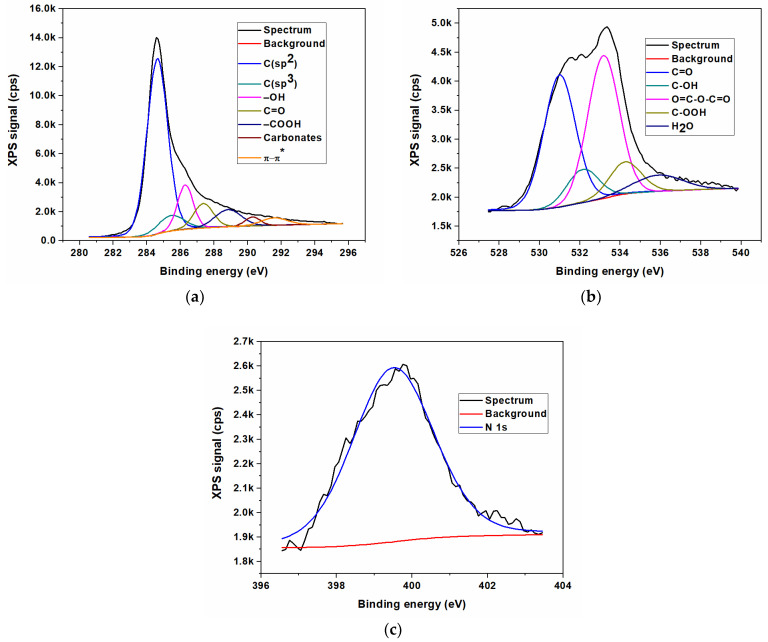
XPS high resolution spectra for nitrogen-doped graphene oxide synthesized using urea (N/GO_U); (**a**) Carbon (C1s); (**b**) Oxygen (O1s); (**c**) Nitrogen N1s.

**Figure 6:**
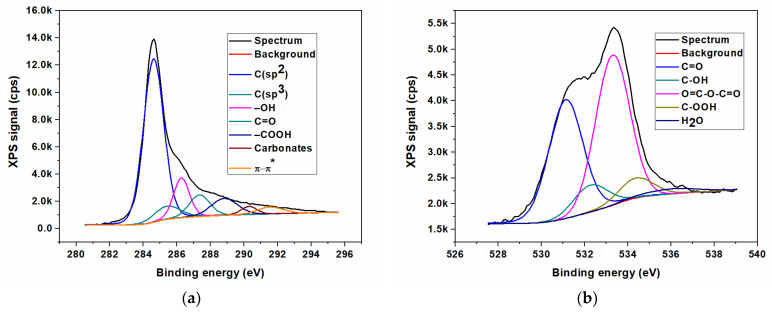

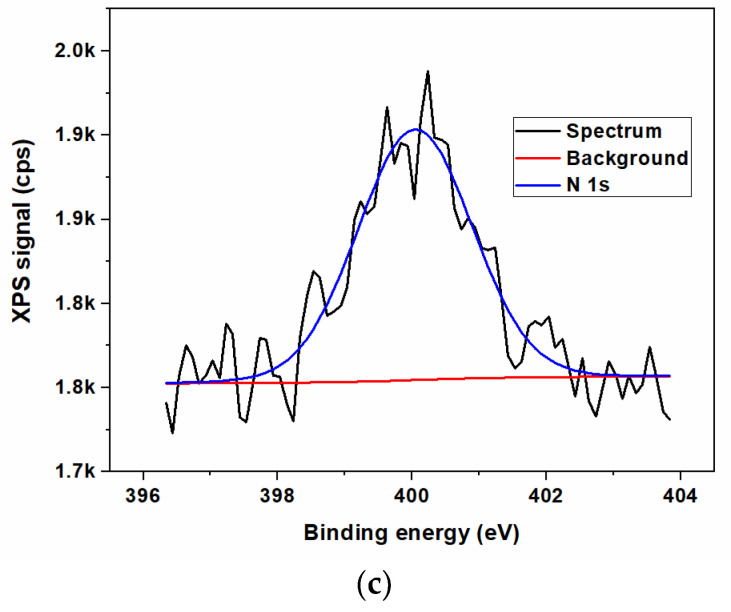
XPS high resolution spectra for nitrogen-doped graphene oxide synthesized using nitric acid (N/GO_N); (**a**) Carbon (C1s); (**b**) Oxygen (O1s); (**c**) Nitrogen N1s.

The XPS was the technique used to investigate the surface composition of prepared materials. The results of X-ray photoelectron spectroscopy measurements, with reference to the non-doped graphene oxide (GO) and to nitrogen-doped graphene oxides, starting from different nitrogen precursors, are presented in Figure 3, Figure 4, Figure 5 and Figure 6. The XPS survey spectra of the prepared materials outlines three major peaks assigned to Carbon (C1s), Nitrogen (N1s), and Oxygen (O1s). The C1s peak indicates an intense peak at 284.5 eV, which correspond to sp^2^ carbon hybridization and carbon atoms single or double bonded to the nitrogen atoms or oxygen. The C1s scan was deconvoluted in three major components located at approx. 284.3, 285.2 and 288.2 eV, assigned to C-C/C=C, C-O/C=O, and C-N/O-C=O bonding [35]. The peak from 284.5 eV was assigned to the graphitic carbon, showing that the most part of the carbon atoms were settled in conjugated honeycomb lattice. The C1s spectra indicated that the N-functionalized carbon where the nitrogen atoms were bonded within C through a graphitic matrix. The high-resolution N1s spectra were deconvoluted into three components: pyrrolic nitrogen (399.2 eV), graphitic nitrogen (399.7 eV), and pyridinic nitrogen (401.6 eV). These components were similar to those described for nitrogen-doped graphene in [35,36]. The O1s oxygen peaks look broad as a result of the doping process, indicating the existence of various chemical states for oxygen. Moreover, the oxygen functionalities for O1s were identified as carbonyl oxygen (531.4 eV), by using the deconvolution of the peaks corresponding to non-carbonyl oxygen atoms from esters (532.5 eV), and oxygen atoms from carboxylic functionality (531.7 eV) [14,37]. The O 1s peak relative to the corresponding C 1s peak observed for nitrogen-doped graphene indicates a stronger O_2_ adsorption, which could be an additional advantage as an ORR electrode for PEM fuel cells.

The presence of large amounts of nitrogen and oxygen atoms on the graphene oxide surface together with the high porosity as will see from BET measurements could provide the necessary adsorption sites for the electrochemical response. Thus, a high amount of N doped in a carbon-based structure could offer the advantage of improving the catalytic and electrochemical properties. The elemental composition of the surface was carried out. The nitrogen content was determined by high resolution XPS measurements and the composition profiles are presented in Table 3.

The atomic concentrations of the chemical elements were determined from the areas of the peaks, taking into account the sensitivity factors of the analyzed elements, thus performing the quantitative elemental analysis. The concentration of nitrogen dopant incorporated into the final samples, according to the peak areas, indicated atomic percentages of 6.7 wt.%, 3.5 wt.%, and 2.2 wt.%, respectively, for the sample N/GO_A, N/GO_U, and N/GO_N. It can be noted the high degree of doping obtained in mild reaction conditions, the highest being for the sample obtained from ammonia as precursor.

Surface area and pore size are factors of most interest in processes involving gas or liquid interacting surfaces. An important characteristic of porous materials is the specific surface area of the materials, commonly calculated from the BET equation. The porous materials having a high specific surface area could offer more active sites and favor the kinetics and transport properties. The nitrogen adsorption and desorption curves were analyzed to estimate the specific surface area. The N_2_ adsorption–desorption isotherms were analyzed and their shapes correspond to type IV in compliance to the IUPAC classification. The vertical gas adsorption in the low-pressure range (up to P/P_0_ = 0.02), as well as the appearance of a pronounced hysteresis loop between P/P_0_ = 0.4 and P/P_0_ = 1.0 are the results of the coexistence of both micropores but also mesopores. The calculated BET area and the estimated textural properties for pore volume and pore radius are shown in Table 4.

It was found that the specific area was smaller than the estimated surface area for the original GO material (421 m^2^ g^−1^), according to [38,39]. The BET values obtained for analyzed materials indicated that the chemical doping process during microwave has caused a decreasing of the specific surface area. The pore volume was diminished in comparison to that of the original GO structure. This indicated that some mesopores have been partially clogged due to the doping process. Thus, slightly lower values were calculated for prepared N/GO, as presented in Table 4.

**Figure 7:**
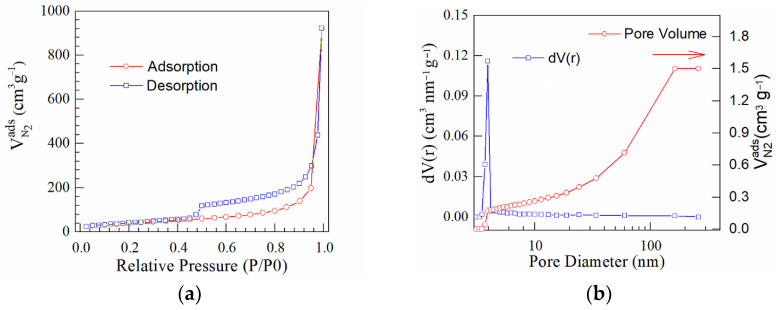
(**a**) N_2_ adsorption–desorption isotherms; and (**b**) BJH curves corresponding to N/GO_A.

**Figure 8:**
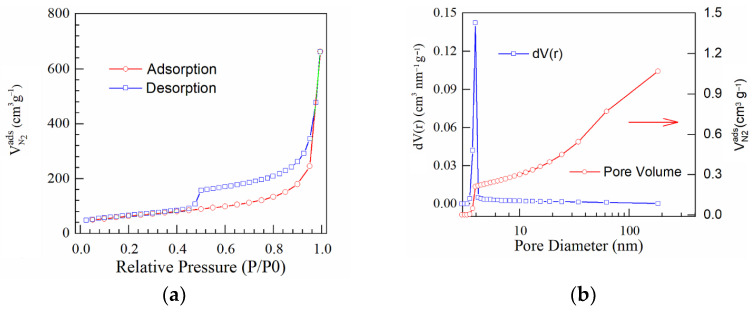
(**a**) N_2_ adsorption–desorption isotherms and (**b**) BJH curves corresponding to N/GO_U.

**Figure 9:**
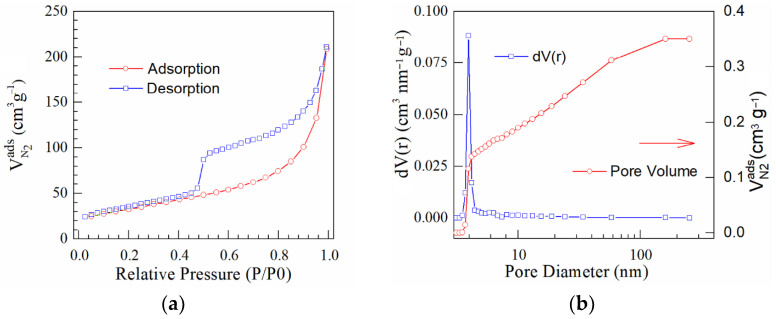
(**a**) N_2_ adsorption–desorption isotherms and (**b**) BJH curves corresponding to N/GO_N.

The nitrogen adsorption–desorption isotherms (Figure 7, Figure 8 and Figure 9) for these materials can be classified as representative for Type IV isotherms. These curves indicated well-defined H3 hysteresis loops which usually are accompanied by the capillary condensation. The difference between the specific surfaces were caused by the doping effect during the microwave process.

The difference noted for the calculated specific surface area (S_BET_) could be correlated with nitrogen content of each sample. Thus, the S_BET_ for N/GO_A and N/GO_N was estimated at 67 m^2^ g^−1^ and 117 m^2^ g^−1^, respectively, while the nitrogen content was 7.42 wt.% and 2.44 wt.% These results indicated that the lowest S_BET_ corresponds to the highest nitrogen content, possibly due to the obtaining of a chemically more doped graphene oxide during the microwave reaction. It was found that the lowest specific surface area corresponds to the GO doped in the presence of ammonia. The hysteresis type indicates the presence of the micro and mesoporous structure in the N/GOs layers with plate-like slit-shaped pores. The pore size distribution calculated by BJH method considering the desorption branch displays a unimodal peak centered at 7 nm. The BJH pore-size distribution method pointed out the dominant role of mesoporosity, which contributed to approx. 65.8% of the total pore volume, while the microporosity contributed to approx. 33.2%. The remaining approx. 1% of the total pore volume was caused by macroporosity.

**Figure 10:**
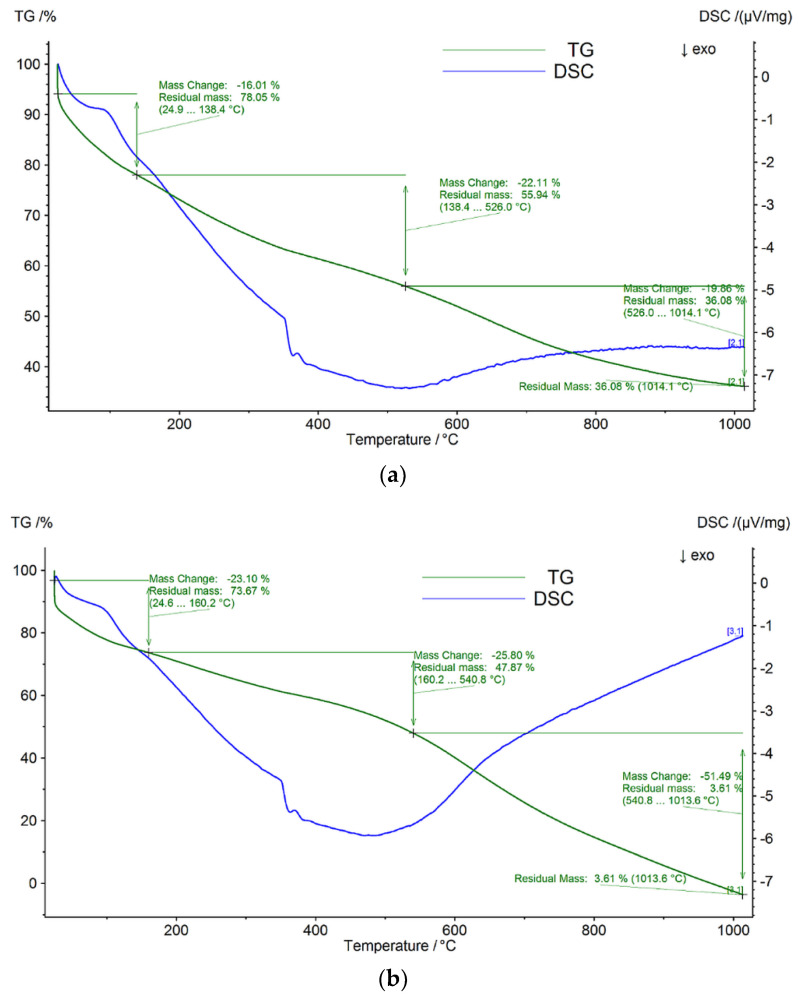

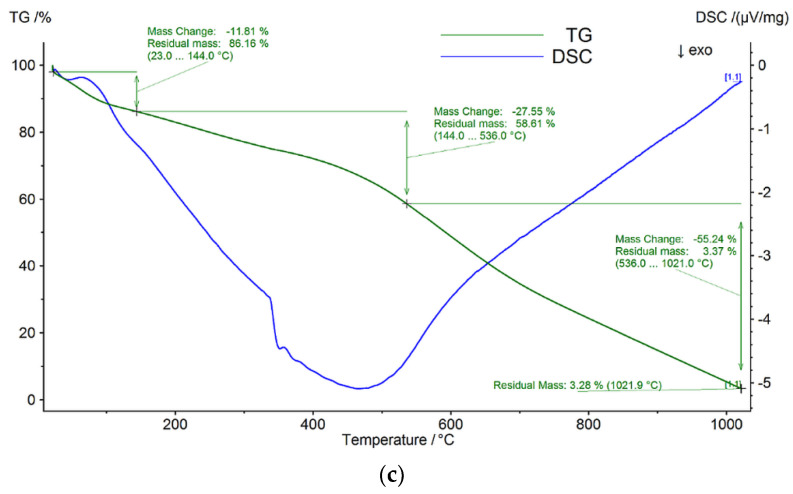
Thermogravimetric analysis (TGA) and differential scanning calorimetry (DSC) measurements of nitrogen-doped graphene oxides. (**a**) TGA and DSC curves corresponding to N/GO_A. (**b**) TGA and DSC curves corresponding to N/GO_U. (**c**) TGA and DSC curves corresponding to N/GO_N.

Thermogravimetric analysis (TGA) was employed to investigate the thermal stability of the prepared nanocomposites. The synthesized nitrogen-doped graphene exhibited similar curves with good thermal stability. TGA weight loss curves of prepared samples are presented in Figure 10. It was observed that the obtained thermograms show different temperature ranges for the thermal decomposition: an important mass loss in the decomposition range of 150–300 °C, emphasizing the efficient removal of oxygen functional groups during the chemical process in the microwave field; a continuous mass loss from 300–600 °C, possibly due to the loss of sp^2^ carbon atoms in a hexagonal structure that occurs between the decomposition temperatures of 320 and 650 °C [40], which is associated with the elimination of certain functionalities specified by FTIR analysis. It is important to note that the drop in the 20–150 °C domain, especially for the sample N/GO_A is certainly due to the high nitrogen content, and is consistent with the elemental analysis in Table 1. Analysis of the thermal stability of N/GOs could be related to the nitrogen bonding configuration (nitrogen functionalities indicated by XPS analysis) in a graphene network, indicating that the pyridinic-N configuration is mostly dominant, instead of graphitic N [41]. According to this reference, the pyridinic-N configuration is mostly dominant at lower doping temperatures. This is precisely the case of the temperature used in actual experimental studies of N/GO preparation, as the working temperature was 60–80 °C during microwave reaction. However, this aspect may indicate that the nitrogen content is related to the process temperature in the microwave field, as well as to the degree of doping. Thus, we can understand that it would be possible to provoke and induce a configured and selective doping with nitrogen atoms, which is a major breakthrough in optimizing and tuning the physicochemical properties of nitrogen-doped graphene materials.

According to the literature, the nitrogen-doped graphene could provide a comparable electrocatalytic activity for the oxygen reduction reaction, and a higher durability and selectivity than the classic expensive Pt-based catalysts. These electrochemical performances were usually related to the nitrogen functional groups, as well as to the specific properties of graphene.

This highly doped nitrogen-doped graphene could exhibit electrochemical activity towards oxygen reduction in alkaline medium providing an affordable industrial alternative to currently used noble metal-based catalyst, such as Pt, Pd [42,43]. Alkaline electrolytes have notable importance in FCs research. Among alkaline electrolytes, the aqueous solution of KOH is of particular interest in PEMFCs operation. Similar to acidic electrolytes, the high ionic conductivity of alkaline electrolytes improves the performance of FCs by engaging functional groups attached to electrode material (e.g., –COOH group in graphene, nitrogen-doped carbon) and electrolyte ions. According to the accepted four-electron associative mechanism for oxygen reduction reaction referring to the nitrogen-doped graphene in alkaline solution, the intermediate OH (ads) is formed from O (ads) with the addition of H_2_O and one electron. It was presumed that the OH (ads) could be attached to the catalytic core as well to the active site through a chemical bond. Thus, the ORR active sites can be determined from the presence of intermediate OH (ads). It is known that the −OH-attached pyridinic nitrogen after the electrochemical reaction suggests that pyridinic N acts an important role in the ORR process and the nearby C atoms represent the main active site, according to existing theoretical and experimental studies [12,43]. Thus, the neighbor C atoms of pyridinic N influence the atomic charges which affect the ORR: the absorption of the intermediate products, the formation of a C–O bond, and the disassociation of an O–O bond.

In order to investigate the electrocatalytic activities of the N/GO, different electrochemical measurements were involved in two stages: the initial measurement (beginning of life BoL) and the final measurement corresponding to the moment when the activity was diminished with 10% (end of life EoL). The catalytic activity for ORR was studied for the samples with the highest nitrogen content by CV carried out in a 0.1 M KOH solution. Before each test, the solution was saturated with oxygen.

**Figure 11:**
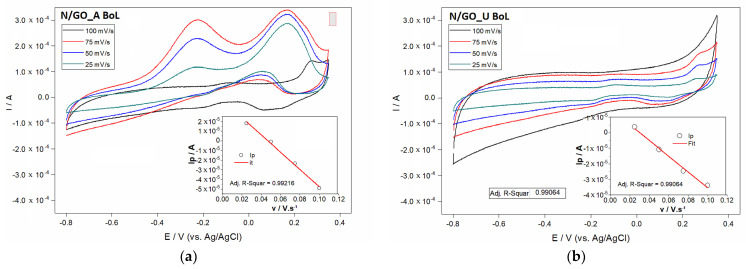
Cyclic voltamograms at different scan rates for N/GO_A (**a**) and N/GO_U (**b**) for BoL. Right bottom insert plot of the dependency of oxidation peak current on the square root of the rate.

The use of various scan rates is a proper methodology in terms of identification of oxidation and reduction peaks. Figure 11 shows the comparison of the voltametric cycles of N/GO_A and N/GO_U at different scan rates. Slight shifts in peak potentials were observed with increasing scan rate indicating some kinetic limitations. Reduction peaks were determined around of +0.15 and +0.2 V for N/GO_A and 0.05 and 0.15 V for N/GO_U, respectively. Peaks identified for oxidation were determined around of −0.25 and +0.2 V for N/GO_A and around of 0.25 V for N/GO_U, respectively. Anodic peak currents were plotted against square root of scan rate, as shown in Figure 11. The linearity of plots (*R*^2^ = 0.99216; *R*^2^ = 0.99064) confirmed that the mass diffusion-controlled electron transfer in all cases.

The most positive ORR peak potential and the highest peak current of N/GO_A towards N/GO_U must be discussed considering the difference of nitrogen content from compared samples. As reported, this content could cause distinct electrochemical properties, because during N doping, some C atoms are replaced, but some defects also appear leading to the structural distortions of GO [44].

Doped nitrogen atoms into the graphenic structure act as electron acceptor sites. The electron-accepting ability of the nitrogen atom can convert the next-neighbor carbon as positive, leading to a redistribution of spin density and charge density in the vicinity of N atoms. These properties influence the oxygen absorption and electrochemical reactions for ORR. As demonstrated, the atomic spin density and charge transfer cause electrocatalytic capacity for ORR.

The composition-dependent ORR activity is influenced by graphitic N and pyridinic N, considered to be responsible for the ORR. The spin and charge density was calculated for the charge transfer and was found that the carbon atoms with charge transfer higher than 0.15 can be used as active sites. A calculation regarding the percentage of C atoms with larger charge density (over 0.15) as function of N content in graphene, indicated that the active sites of C atoms increase linearly as the N content in graphene grows and the maximum value of charge transfer also increase with the N content from 0.16 for 8% [44]. Thus, the sample N/GO_A with the nitrogen content of 7.2% have more catalytic reactive sites than N/GO_U and leads to a relatively better catalytic capability for ORR, which is consistent with our experimental observations.

Figure 11 shows the CV of oxygen reduction as a large cathodic peak current, demonstrating the activity of the both samples at the beginning of life moment. Both nitrogen-doped graphene showed a good onset potential, being known the fact that the onset potential indicates the catalytic activity of the catalysts. N-doping of graphene leads to the significant improvement of its functional characteristic in particular the electrocatalytic activity in oxygen reduction reaction (ORR), which is current-forming process in fuel cells. Well-defined cathode peaks were obtained for different scan rate. The electrochemical properties might be attributed to the introduction of defects and nitrogen-containing groups at the electrode surface, which could accelerate the charge transfer rate across electrode and ions solution interface. The comparison between N/GO_A and N/GO_U indicates the highest current for N/GO_A sample. It is quite clear that the oxidation peak and reduction current corresponding to the N/GO_A electrode are noticeably higher than those of the N/GO_U electrode, indicating that the first sample significantly improves the electrochemical kinetics of the redox reaction in air-saturated 0.1 M KOH electrolyte.

**Figure 12:**
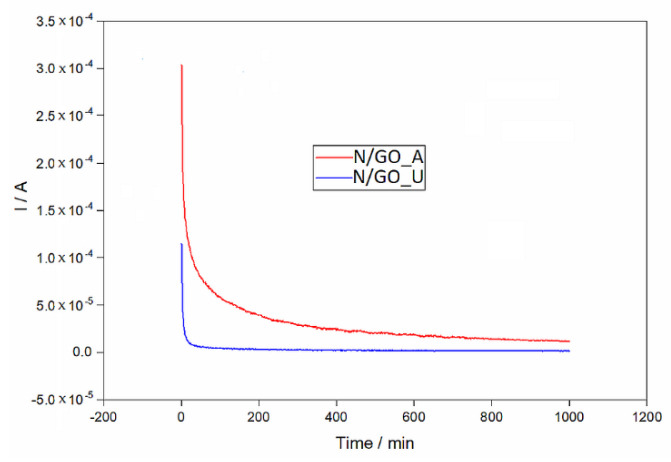
Chronoamperometric curves of different catalysts synthesized in the present work in 0.1 M KOH electrolyte at a constant potential of 0.3 V for 1000 min.

The long-term stability of the prepared samples was evaluated by chronoamperometry. The experiments were performed at a fixed potential of 0.2 V in the saturated KOH solution for 1000 min at the room temperature and the obtained data are presented in Figure 12. Both catalysts indicated a constant current decay. The current decreased more slowly for the N/GO_A electrode than for the N/GO_U electrode, indicating probably a less accumulation of adsorbed poisoning species.

**Figure 13:**
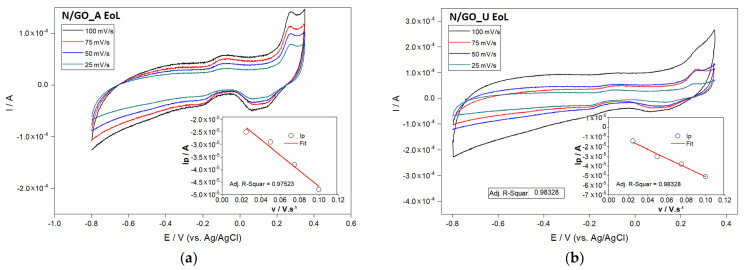
Cyclic voltamograms at different scan rates for N/GO_A (**a**) and N/GO_U (**b**) for EoL. Right bottom insert plot of the dependency of the oxidation peak current on the square root of rate.

The investigation using CV analysis after the stability test was taken into account in order to evaluate the electrochemical activity of the depleted catalyst. The end-of-life results are presented in Figure 13. Here the linear variation of the peak current with the square root of the scan rate for both N/GO catalysts is depicted in the insets.

The smallest peak potential difference (ΔEsep) was observed for the N/GO_A (Figure 13). Consequently, both the electrochemical activities and the reversibility were more efficient, probably due to the improved electrochemical properties, which could be attributed to the introduction of defects and nitrogen-containing groups at the electrode surface, accelerating the charge transfer rate across solution interface.

**Figure 14:**
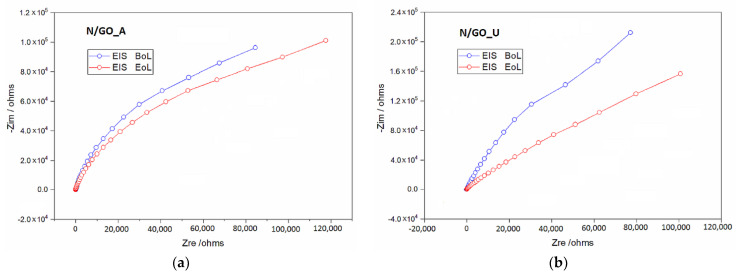
Nyquist plots of the catalysts N/GO_A (**a**) and N/GO_U (**b**).

To investigate the electrochemical performance of the proposed N/GO electrode, the electrical impedance spectroscopy (EIS) was carried out at open-circuit potential. The Nyquist plot is presented in Figure 14. It was observed that the obtained curves include a linear part in the frequency between 0.01 Hz and 10 kHz, which suggests a mass transfer process. Moreover, these are an indication that both charge transfer and mass diffusion play an important role in the redox reaction. This behavior may be because the lone pair electron caused by the doped nitrogen improves the electrical conductivity of the electrode and reduces the electrochemical polarization during the redox reaction.

Performance in the PEMFC represents the key viability test for graphene-based PGM-free catalysts, due to the fact that the catalyst must facilitate the gas and proton transport through the cathode thickness and with graphene microstructure to the active sites, which are properties not provided by ex situ measurements.

Based on the structural characterization presented above, one of the prepared N/GOs (catalyst with the highest nitrogen concentration) was tested under practical fuel cell operation conditions, using a single FC with an active area of 25 cm^2^. A set of PEMFC measurements were carried out to analyze the cathode performance, following the common benchmarking practice. After achieving steady-state operating conditions, the polarization curve was evaluated. The experiments were performed in potentiostatic mode and a predefined protocol was applied.

Thus, using an N/GO_A -based membrane electrode assembly (MEA), with the highest power density achieved at 0.5 V was 343 mW cm^−2^ (720 mA cm^−2^) and current density achieved at 0.7 V was 315 mA cm^−2^.

The performance tests were carried out in potentiostatic mode by sweeping the voltage from OCV to the final voltage of 0.476 V with 8 mV s^−1^. The test parameters were the following: temperature in the range of 61–62 °C, atmospheric pressure at the anode/cathode, stoichiometry 1.2 and 2.5 at anode and cathode, respectively. The following were monitored: the produced current, the flow rates of reactants and the dew point temperature.

**Figure 15:**
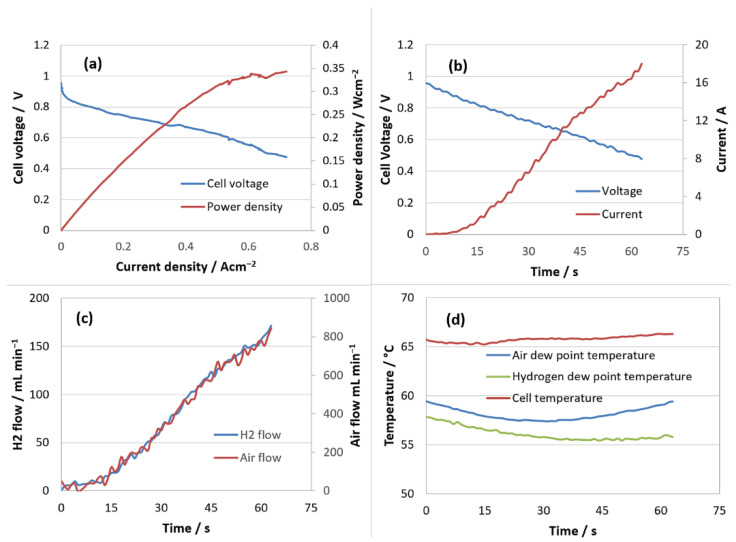
PEMFC performance using N/GO_A -based MEA. (**a**) Polarization and power density curves; (**b**) current variation profile depending on voltage; (**c**) flow rates consumed; (**d**) profiles: temperature of the PEMFC with MEA based on nitrogen doped graphene, and dew points of the reactants.

A cyclic load profile has been proposed in order to investigate the electrocatalyst activity, as shown in Figure 15. A stress parameter was considered, in respect to the fuel cell sweeping voltage, varying between OCV and 0.476 V to accentuate the degradation of the fuel cell. The reported current density at 0.7 V in these operation conditions is comparable or even higher than state-of-the-art results related to PGM-free PEMFC [45,46,47].

The above results demonstrate good performance of PEMFC using N/GO-based MEA. N/GO could be a promising candidate for practical application in cathode electrode under single FC working conditions. The presence of N/GO can effectively enhance the electrocatalytic stability for ORR. In this regard, N contributes as spacer between interconnected 3D porous structure avoiding the GO sheets restacking, favoring the transportation of reactants and product and also could provide many accessible active sites with electrocatalytic stability.

## 4. Conclusions

N/GO catalyst emerges as one of the promising non-PGM catalysts with the advantages of low cost and high ORR catalytic performance to replace expensive PGM catalysts in electrochemical systems. This work introduces a novel process for preparing of nitrogen-doped graphene by using microwave treatment under ambient conditions, as simple, effective, faster and economical protocol. The physical and chemical properties of the materials obtained from different precursors (ammonia, urea and nitric acid) were evaluated and compared and it was found that ammonia allows to obtain a higher concentration of nitrogen, due to its high vapor pressure which facilitates the functionalization reaction of GO during microwave. N/GO was obtained with the highest nitrogen concentration of 7 wt%, a high specific surface and a high porosity which gives the potential as catalytic material. The BET evaluation suggests that the doping process led to a slight decrease in the specific surface, but the pore volume was quite well preserved compared to the original graphene structure. This paper indicates that nitrogen-doped graphene with a high dopant level could be prepared by starting from a commercially accessible material—graphene oxide, as raw material, through an optimized designed process for chemical synthesis under the microwave field. It is believed that this approach can bring many possibilities in tailoring the chemical properties. Moreover, this route could be a step in the development of other types of non-metallic graphene-based catalysts by using MW. Furthermore, considering this cost-effective strategy, it is expected to broaden N/GO beyond fuel cell application.

## Figures and Tables

**Table 1:** Nitrogen-doped graphene oxide obtained by using the microwave method; comparison of N doping ratio based on experimental condition (inorganic N precursors: ammonia and nitric acid).

Sample N/Gr	Ammonia (mL)	Nitric Acid (mL)	Reduction Agent	T (°C)	C % (wt.)	N % (wt.)	H % (wt.)	O % (wt.)
**1**	**44**	**-**	**EG**	**80**	**78.98**	**7.52**	**0.94**	**12.56**
2	40	-	EG	80	79.35	6.96	0.98	12.71
3	36	-	EG	80	80.6	6.68	0.93	11.79
4	44	-	EG	60	82.41	5.2	1.16	11.23
5	40	-	EG	60	81.99	4.82	1.28	11.91
6	36	-	EG	60	82.58	4.18	1.26	11.98
7	44	-	BH_4_Na	80	81.31	5.56	1.07	12.06
8	40	-	BH_4_Na	80	80.87	5.45	1.12	12.56
9	36	-	BH_4_Na	80	80.99	5.41	1.18	12.42
10	44	-	BH_4_Na	60	82.57	5.09	0.96	11.38
11	40	-	BH_4_Na	60	82.59	4.62	1.22	11.57
12	36	-	BH_4_Na	60	82.71	4.21	1.36	11.72
13	44	-	Et	80	80.16	5.63	1.16	13.05
14	40	-	Et	80	79.92	5.58	1.24	13.26
15	36	-	Et	80	79.94	5.29	1.28	13.49
16	44	-	Et	60	80.42	4.65	1.19	13.74
17	40	-	Et	60	80.44	4.43	1.21	13.92
18	36	-	Et	60	80.59	4.16	1.23	14.02
**19**	**-**	**44**	**BH_4_Na**	**80**	**82.12**	**2.68**	**1.57**	**13.63**
20	-	40	BH_4_Na	80	82.55	2.44	1.73	13.28
21	-	36	BH_4_Na	80	81.77	2.23	1.78	14.22
22	-	44	BH_4_Na	60	81.51	1.98	1.83	14.68
23	-	40	BH_4_Na	60	81.73	1.81	1.87	14.59
24	-	36	BH_4_Na	60	81.96	1.33	1.93	14.78

**Table 2:** Nitrogen-doped graphene oxide materials obtained by using the microwave method; comparison of N doping ratio based on experimental condition (organic nitrogen precursor: urea).

Sample N/Gr	Urea (g)	Reduction Agent	T (C)	C % (wt)	N % (wt)	H % (wt.)	O % (wt.)
**1**	**3**	**EG**	**80**	**82.57**	**3.89**	**1.25**	**12.29**
2	2.5	EG	80	82.42	3.63	1.28	12.67
3	2	EG	80	82.18	3.59	1.31	12.92
4	3	EG	60	81.84	3.71	1.27	13.18
5	2.5	EG	60	81.73	3.62	1.29	13.36
6	2	EG	60	81.71	3.38	1.32	13.59
7	3	BH_4_Na	80	81.95	2.25	1.38	14.42
8	2.5	BH_4_Na	80	82.40	1.93	1.39	14.28
9	2	BH_4_Na	80	82.19	1.76	1.42	14.63
10	3	BH_4_Na	60	81.95	1.84	1.49	14.72
11	2.5	BH_4_Na	60	81.88	1.72	1.52	14.88
12	2	BH_4_Na	60	81.94	1.54	1.56	14.96
13	3	Et	80	83.61	3.06	1.26	12.07
14	2.5	Et	80	83.39	2.95	1.29	12.37
15	2	Et	80	83.51	2.74	1.33	12.42
16	3	Et	60	83.40	2.91	1.41	12.28
17	2.5	Et	60	83.32	2.77	1.46	12.45
18	2	Et	60	83.46	2.56	1.47	12.51

**Table 3:** XPS quantitative analysis—composition profile.

Element	GO	
	Weight%	Atomic%
C1s	82.9	86.61
O1s	17.09	13.39
**Element**	**N/GO_A**	
	**Weight%**	**Atomic%**
C1s	76.93	80.96
O1s	15.64	12.34
N1s	7.42	6.7
**Element**	**N/GO_U**	
	**Weight%**	**Atomic%**
C1s	79.73	83.62
O1s	16.47	12.88
N1s	3.8	3.5
**Element**	**N/GO_N**	
	**Weight%**	**Atomic%**
C1s	80.29	84.22
O1s	17.26	13.58
N1s	2.44	2.2

**Table 4:** Textural properties of nitrogen-doped graphene oxides.

Samples	S_BET_ (m^2^ g^−1^)	BJH Pore Volume (cm^3^ g^−1^)	BJH Pore Radius (A)
GO	421	1.629	19.691
N/GO_A	67	0.196	19.644
N/GO_U	83	0.268	19.675
N/GO_N	117	0.355	19.678

## Data Availability

The authors confirm that the data supporting the findings of this study are available within the article. Raw data that support the findings of this study are available from the corresponding author, upon reasonable request.
